# The Stress−Dilatancy Behaviour of Artificially Bonded Soils

**DOI:** 10.3390/ma15207068

**Published:** 2022-10-11

**Authors:** Zenon Szypcio, Katarzyna Dołżyk-Szypcio

**Affiliations:** Department of Geotechnics and Structural Mechanics, Faculty of Civil Engineering and Environmental Sciences, Bialystok University of Technology, Wiejska 45E, 15-351 Białystok, Poland

**Keywords:** stress–dilatancy, artificially bonded soils, frictional state concept

## Abstract

In this study, the results of triaxial compression tests of some naturally and artificially bonded soils presented in the literature were analysed. It was shown that the three characteristic stages of plastic flow during shear can be identified. In all stages, the stress–dilatancy behaviour could be approximated by the general linear stress–dilatancy equation of the Frictional State Concept. For many shear tests, the failure states and newly defined dilatant failure states are not identical. The points representing dilatant failure states lie on a straight line, for which the position and slope in the η-D plane depend on the soil type and the amount of cement admixture. This line defines the critical frictional state angle, and its slope for bonded soils is greater than for unbonded soils.

## 1. Introduction

Bonded natural soils have high strength and stiffness and can be suitable subsoils for dams and high-rise buildings. Bounding (cementation) is the result of physical–electro–chemo–bio interactions between individual grains. The level of cementation in natural soils is a function of grain mineralogy, temperature, the stress level during formation, age, weathering conditions, and many other factors influencing the magnitude of forces necessary to reposition the grains in relation to each other [1,2]. Similar properties to structural natural soils can be obtained by using various additives to induce or increase inter-grain cementation in the case of weak soils. The most commonly used additives are Portland cement, gypsum, lime, fly ash, bacteria, etc. [3,4,5,6]. Naturally bonded (cemented) soils, even formed and weathered in similar conditions, are usually anisotropic and have different physical and mechanical properties [7,8]. The structure of natural soils is partially destroyed during sampling, transportation, and sample preparation for laboratory tests, which is the main difficulty in determining the physical and mechanical properties of these soils [9,10]. Many researchers, e.g., [7,10], have shown that the behaviours of naturally and artificially bonded soils are similar. Therefore, tests of artificially bonded soils are most often performed [11,12]. The behaviour of bonded soils is significantly more complex than that of soils with unbonded grains. Many constitutive models for soils take the critical state as reference state [13,14]. For bonded soils, the critical state is difficult to achieve in a triaxial compression test. For a low-stress level, the shear band is formed, the deformation of the sample is very inhomogeneous, and the stresses and strains are very difficult to calculate correctly. For a very high-stress level, the critical state is not reached with high shear strains. Some constitutive models for structured soils are based on the disturbed state concept [15].

Stress–dilatancy behaviour is very important in soil modelling and depends on the deformation mode, stress level, and stress path [16]. The stress–dilatancy relationship of unbonded soils can be approximated by straight lines in different stages of shearing, defined by the critical frictional state angle (φ°) and two (α and β) parameters of the frictional state concept [17,18]. Contrary to what was observed in unbonded soils, the maximum dilatancy was reached beyond the maximum strength for bonded soils [19,20].

In this paper, the stress−dilatancy relationships are calculated for some artificially bonded soils tested under axial symmetry conditions presented in the literature. The points representing newly defined dilatant failure states lie on a straight line termed the dilatant failure line. The position and inclination of that line in the η−D plane depend on the amount of cement additives.

## 2. General Stress−Plastic Dilatancy Equation

The general stress–plastic dilatancy equation for axial symmetry compression has a simple form [17]:

(1) $$\eta =Q-A\;D^{p}$$

where:

η=q/p′;

$Q=M_{c}^{o}-\alpha A_{c}^{o}$;

$A=\beta A_{c}^{o}$;

$D^{p}=\delta \varepsilon_{\upsilon }^{p}/\delta \varepsilon_{q}^{p}$;

$M_{c}^{o}=6\sin\phi^{o}/\left(3-\sin\phi^{o}\right)$;

$A_{c}^{o}=1-M_{c}^{o}/3$, for drained triaxial compression;

$A_{c}^{o}=1+2M_{c}^{o}/3$, for undrained triaxial compression;

$q=\sigma_{1}^{\prime }-\sigma_{3}^{\prime }$;

$p^{\prime }=\left(\sigma_{1}^{\prime }+2\sigma_{3}^{\prime }\right)/3$;

$\delta \varepsilon_{\upsilon }^{p}=\delta \varepsilon_{1}^{p}+2\delta \varepsilon_{3}^{p}=\delta \varepsilon_{\upsilon }-\left(\delta p\prime /K\right)$;

$\delta \varepsilon_{q}^{p}=\frac{2}{3}\left(\delta \varepsilon_{1}^{p}-\delta \varepsilon_{3}^{p}\right)=\delta \varepsilon_{q}-\left(\delta q/3G\right)$;

K—bulk modulus;

$G=3K\left(1-2\nu \right)/2\left(1+\nu \right)$—shear modulus;

ν—Poison’s ratio;

$\delta \varepsilon_{1}$, $\delta \varepsilon_{3}$, $\delta \varepsilon_{1}^{p}$, $\delta \varepsilon_{3}^{p}$—global and plastic parts of maximum and minimum major strain increments, respectively;

$\delta \varepsilon_{\upsilon }$, $\delta \varepsilon_{q}$, $\delta \varepsilon_{\upsilon }^{p}$, $\delta \varepsilon_{q}^{p}$—global and plastic parts of volumetric and shear strain increments, respectively.

For purely frictional shear deformation, α = 0 and β = 1 [18]. The α and β parameters represent the combined influence of destructuration, breakage, and other effects on the stress−dilatancy relationship during shear.

## 3. Stress–Strain Behaviour of Cemented Sand in Triaxial Compression

### 3.1. Characteristic Stages of Shearing

The mechanical behaviour of artificially cemented Portaway sand at high pressures was investigated by Marri [10]. The results of two drained triaxial compression tests of Portaway sand with a 5% Portland cement content at confining pressures $\sigma_{c}=1\;\mathrm{MPAa}$ and $\sigma_{c}=4\;\mathrm{MPAa}$ were analysed in detail to determine the characteristic stages of shearing. The relationships $q-\varepsilon_{a}$ and $\varepsilon_{\upsilon }-\varepsilon_{a}$ obtained in the tests were sectionally approximated by a high degree of polynomials. The elastic parts of the strain increments were calculated using the formula:

(2) $$G=G_{0}\frac{{\left(2.97-e\right)}^{2}}{\left(1+e\right)}{\left(\frac{p'}{p_{a}}\right)}^{0.7},$$

for the shear modulus with $G_{0}=120$, atmospheric pressure $p_{a}=101\;\mathrm{kPa}$ and

(3) $$K=2G\left(1+\nu \right)/3\left(1-2\nu \right),$$

for the bulk modulus. Poisson’s ratio ν=0.25 was assumed for the calculation. The dependence of the bulk modulus on stress for the tested cemented sand with the assumed shear modulus formula (2) and Poisson’s ratio is similar to that obtained in [10].

**Figure 1:**
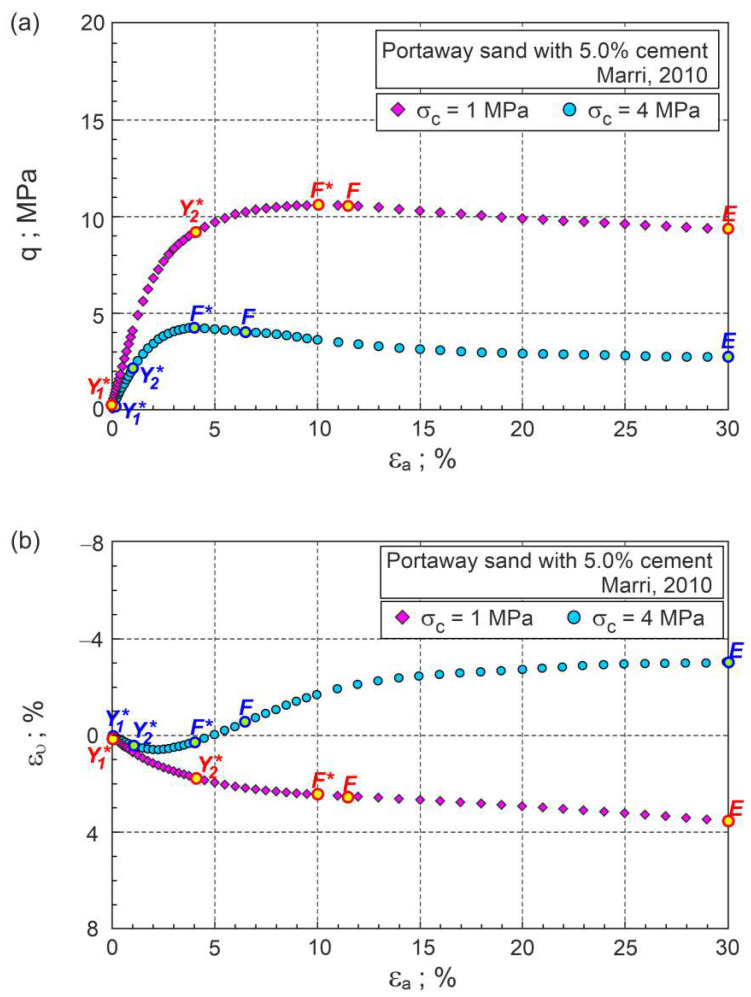
Drained triaxial compression test results for Portaway sand with 5% cement: (**a**) q versus $\varepsilon_{a}$; (**b**) $\varepsilon_{\upsilon }$ versus $\varepsilon_{a}$ (description in the text).

**Figure 2:**
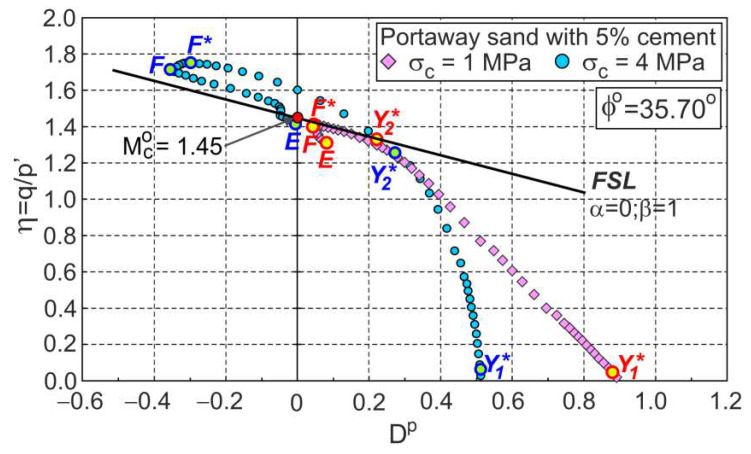
The stress ratio–plastic dilatancy relationship for the drained triaxial compression of Portaway sand with 5% cement.

**Figure 3:**
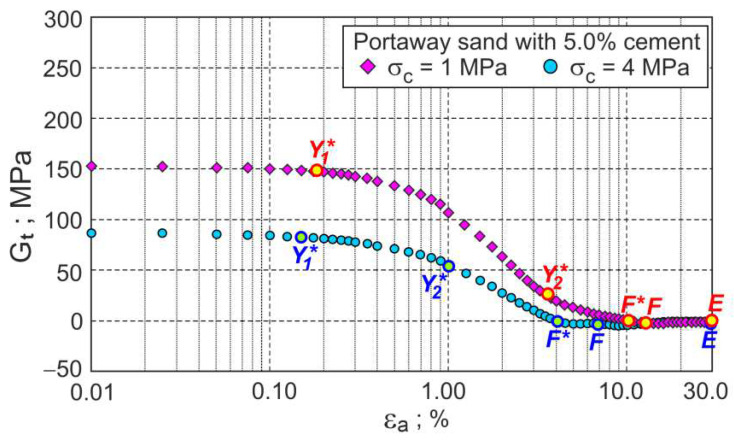
Tangent shear modulus versus axial strain for the drained triaxial compression of Portaway sand with 5% cement.

The approximate experimental values of shear stress (q) and volumetric strains ($\varepsilon_{\upsilon }$) as a function of axial strains are shown in Figure 1. The relations between the stress ratio and plastic dilatancy ($\eta -D^{p}$) and the tangential shear modulus ($G_{t}=\delta q/3\delta \varepsilon_{q}$) as a function of axial strain are presented in Figure 2 and Figure 3, respectively.

During shear, the elastic stage and three plastic flow stages can be distinguished. The points shown in Figure 1, Figure 2 and Figure 3 represent the boundaries between characteristic stages, marked as $Y_{1}^{*}$, $Y_{2}^{*}$, and F, respectively. The E points represent the ends of the tests. The tangent shear modulus must be almost constant at the initial elastic stage due to a small change in the soil void ratio and stresses. Therefore, point $Y_{1}^{*}$ can easily be determined as the point at which the value of the tangential shear modulus begins to decrease (Figure 3). This point corresponds to the $Y_{2}$ point defined by Jardine [21] and the gross yield point in [22,23,24]. For the analysed drained triaxial compression tests of cemented sand under high confining pressures, the $Y_{1}^{*}$ points almost coincide with the initial shear points ($\varepsilon_{a}\approx 0.11\%$) in Figure 1 and Figure 2.

During further shear, the tangent shear modulus gradually decreases and reaches a zero value in the failure state (F* point). During this phase of plastic flow, the rate of the decrease in the tangential shear modulus varies, increasing in the first phase and decreasing in the second phase. Point $Y_{2}^{*}$ represents the shear stage for which the change in the rate of the tangential shear modulus takes place (Figure 3). Point $Y_{2}^{*}$ represents the shear state for which there is a local maximum change in the slope of the $\eta -D^{p}$ curve (Figure 2) and divides this phase of shearing into the first and second stages of plastic flow. In the first stage of the plastic flow, the intensive breakdown of the bonds between grains takes place. In the second stage of the plastic flow, the breakdown of the bonds continues, and intensive changes in the grains’ fabric take place. The maximum effect of destructuration on the shear strength of the bonded soils is observed in the failure state, represented by the F* point. The maximum curvature of the curve $\eta -D^{p}$ (Figure 2) represents the characteristic shear state, called the dilatant failure state, marked as point F in Figure 2. For some shear deformations of bonded soils, the states of failure and dilatant failure are slightly different, e.g., [3,10,19]. A dilatant failure state can be easily identified for the dilative and contractive behaviour of bonded soils, as shown in Figure 2 for cemented Portaway sand. For unbonded granular soils without breakage, the failure and dilatant failure states are identical [18].

In the third plastic flow stage, after the dilatant failure stage, the bonded soil deforms similarly to the unbonded granular material with crushable and various sizes and shapes of the individual grains or their clusters. For most drained triaxial tests of bonded soils, the critical states cannot be reached, such as for the analysed two tests of cemented Portaway sand (Figure 1). The analysed tests were aborted at the axial strain $\varepsilon_{a}=30\%$ and marked as E points in Figure 1, Figure 2 and Figure 3.

### 3.2. Stress–Dilatancy Relationship

**Figure 4:**
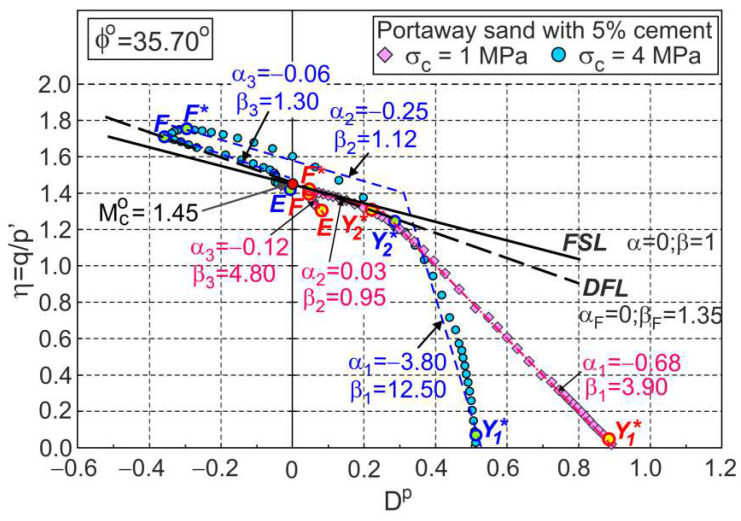
Linear approximations of stress–plastic dilatancy relationships for drained triaxial compression tests of Portaway sand with 5% cement.

As in unbonded granular soils [25,26], the stress–plastic dilatancy relationship can be expressed by Equation (1) for cemented soils. For each of the three (i = 1, 2, 3) stages of plastic shear flow, $\alpha_{i}$ and $\beta_{i}$ can be determined (Figure 4) as the parameters of the linear approximation of the experimental relationships, $\eta -D^{p}$ (Figure 4).

The values of these parameters are a function of the initial structure of the soil and stress level. For the drained triaxial compression tests under high pressures of cemented Portaway sand, the share of the elastic parts of the strain increments in the global strains is very small, and it may be assumed that the plastic dilatancy is equal to the dilatancy $(D^{p}=D$).

### 3.3. Dilatant Failure Line

**Figure 5:**
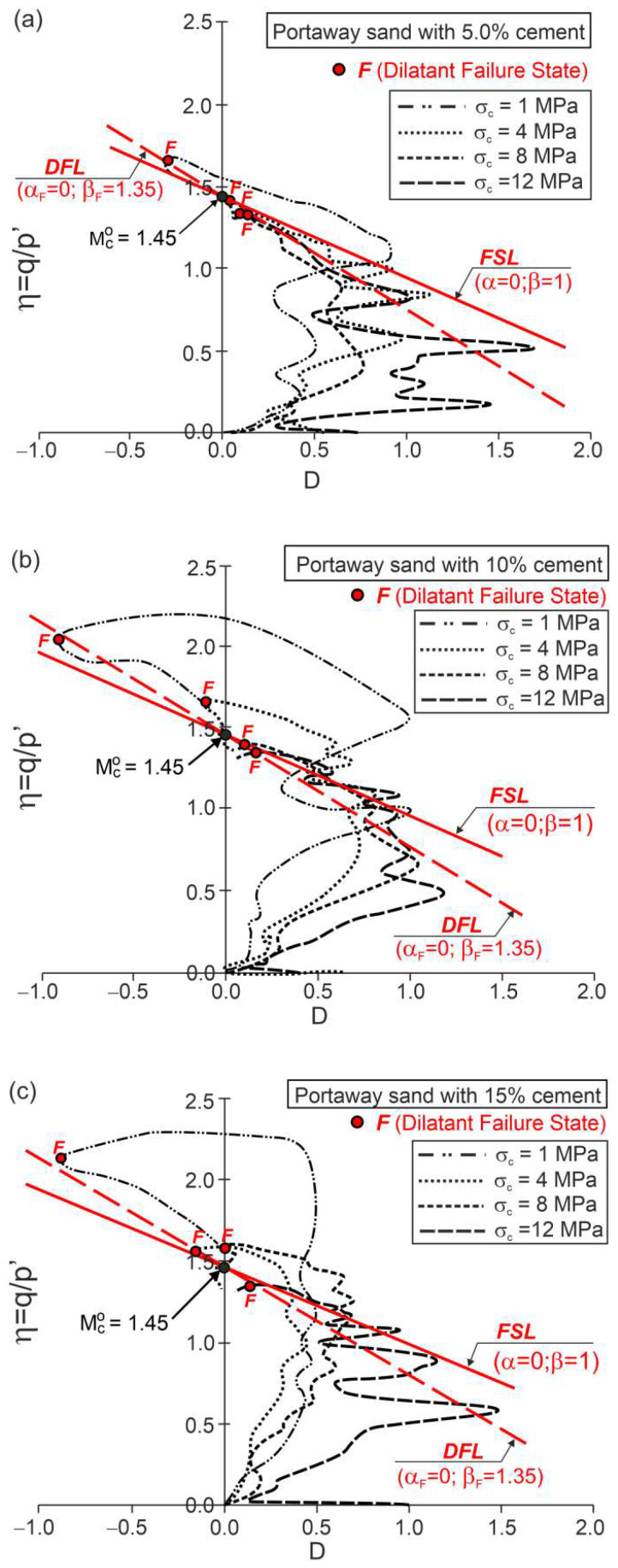
Stress ratio–dilatancy relationships for the drained triaxial compression of cemented Portaway sand: (**a**) 5% cement; (**b**) 10% cement; (**c**) 15% cement (adapted from [10]).

The F points representing the dilatant failure states for the drained triaxial compression tests of cemented Portaway sand with a varying Portland cement content lie on straight lines (Figure 5), called dilatant failure lines (DFLs).

These lines are given by Equation (1) with $\alpha =\alpha_{F}=0$ and $\beta =\beta_{F}=1.35$, independent of the cement content. These DFLs intersect the vertical axes for values of $\eta =M_{c}^{o}=1.45$. Therefore, the critical frictional state angle $\phi^{o}=35.7{}^{\circ}$ is independent of the cement content. The η−D relationships shown in Figure 5 were calculated directly from the experimental data of the stresses and strains and are not as smooth as those shown in Figure 2, which were obtained from approximate data. For clarity, only the F points are shown in Figure 5.

**Figure 6:**
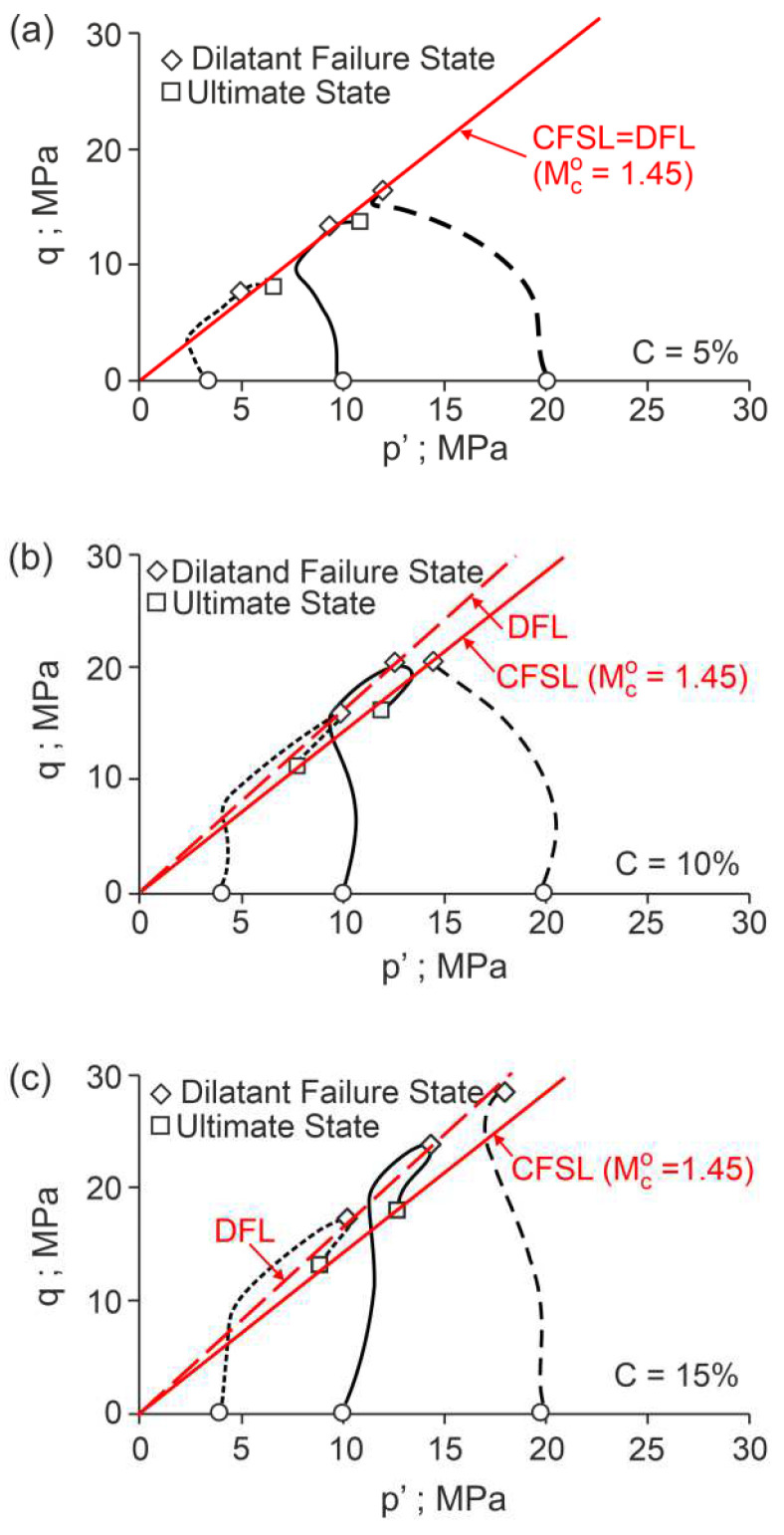
Stress paths for the undrained triaxial compression of cemented Portaway sand: (**a**) 5% cement; (**b**) 10% cement; (**c**) 15% cement (adapted from [10]).

For the undrained triaxial compression tests, the ultimate states (Figure 6) correspond to the critical frictional states ($M_{c}^{o}=1.45$) defined by the intersecting DFL with the vertical axes in the η−D plane for the drained triaxial compression tests (Figure 5). The dilatant failure states are represented by characteristic points of the stress path in q−p′ planes (Figure 6) for the undrained triaxial compression tests. Parameters $\alpha_{F}$ and $\beta_{F}$ could not be determined based on the data shown in [10].

## 4. Dilatant Failure of Some Artificially Bonded Soils

### 4.1. Osorio Sand with Cement Admixture

**Figure 7:**
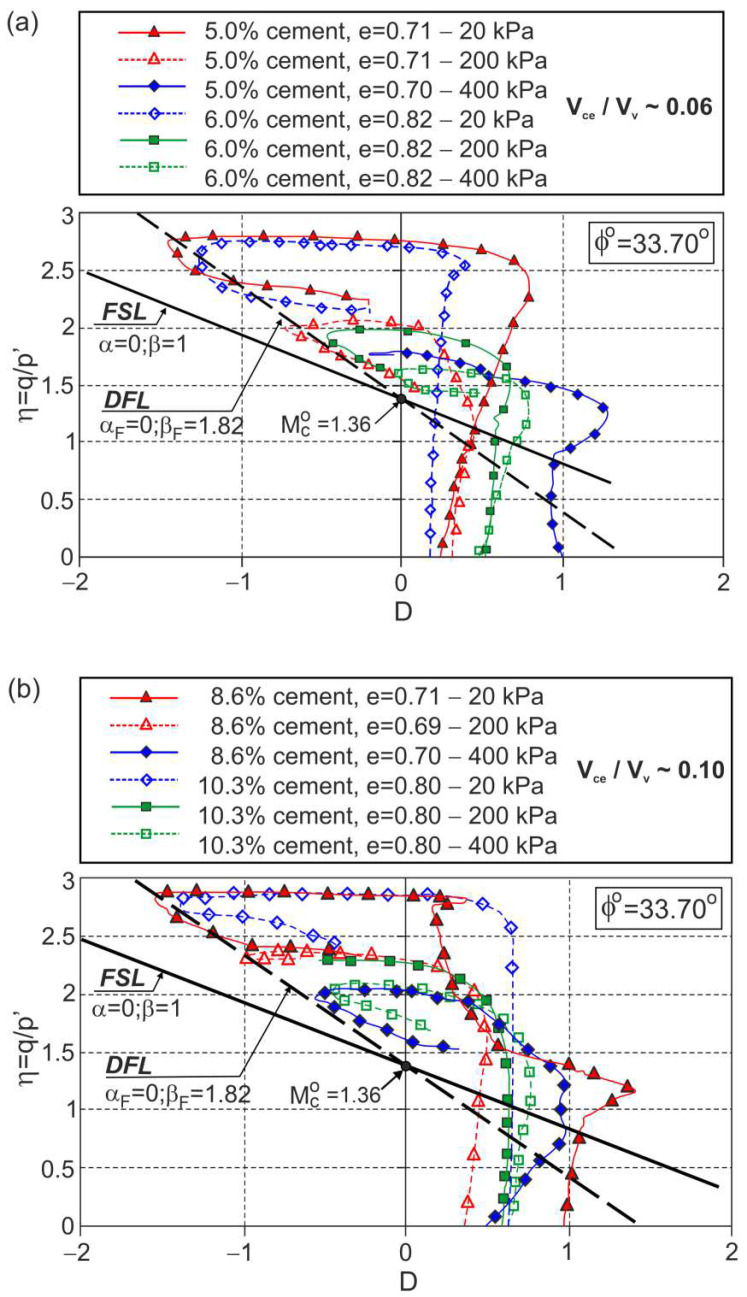
Stress ratio–dilatancy relationships for the drained triaxial compression of fine sand with different cement–void ratios: (**a**) V_ce_/V_v_≈0.06; (**b**) V_ce_/V_v_≈0.10 (adapted from [27]). Used with permission of the American Society of Civil Engineers, ASCE, from Influence of Cement–Voids Ratio on Stress-Dilatancy Behaviour of Artificially Cemented Sand., Consoli, N. C.; Cruz, R. C.; Viana da Fonseca, A.; Coop, M. R., J. Geotech. Geoenviron. Eng. 138 (1), 2012, [doi:10.1061/(ASCE)GT.1943-5606.0000565]; permission conveyed through Copyright Clearance Center, Inc.

Many conventional drained triaxial tests have been carried out on non-plastic, uniform, fine, predominantly quartz Osorio sand with varying amounts of Portland cement by Consoli et al. [27]. The influence of the amount of cement and porosity in the cement−void ratio (V_ce_/V_v_) was investigated—defined as the ratio of the cement volume to the void volume in the mixture—with respect to the stress−dilatancy behaviour. The obtained stress−dilatancy relationships are shown in Figure 7.

For all the tests, three stages of plastic flow are visible. The characteristic points ($Y_{1}^{*}$, $Y_{2}^{*}$, F*, F, and E) are not shown in Figure 7 for clarity. The dilatant failure states can be easily identified, and the approximate DFL can be drawn in the η−D plane. The DFLs for both cement−void ratios $V_{ce}/V_{v}\approx 0.06$ and $V_{ce}/V_{v}\approx 0.10$ are defined by Equation (1) with $M_{c}^{o}=1.36$ ($\phi^{o}=33.7{}^{\circ}$), $\alpha_{F}=0$, and $\beta_{F}=1.82$ (Figure 7).

**Figure 8:**
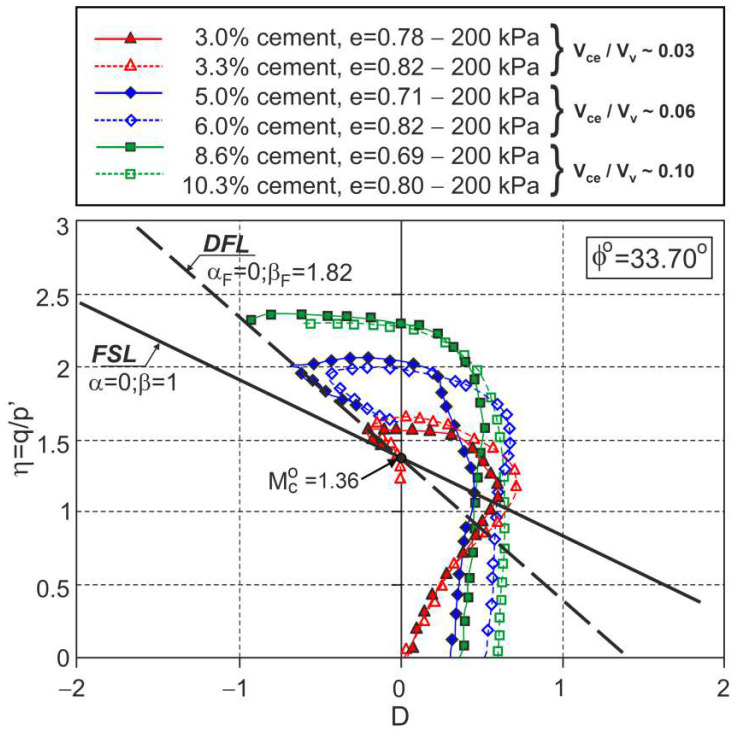
Stress ratio–dilatancy relationships for the drained triaxial compression of fine sand with different volumes of cement and different cement–void ratios (adapted from [27]). Used with permission of the American Society of Civil Engineers, ASCE, from Influence of Cement–Voids Ratio on Stress-Dilatancy Behaviour of Artificially Cemented Sand., Consoli, N. C.; Cruz, R. C.; Viana da Fonseca, A.; Coop, M. R., J. Geotech. Geoenviron. Eng. 138 (1), 2012, [doi:10.1061/(ASCE)GT.1943-5606.0000565]; permission conveyed through Copyright Clearance Center, Inc.

The same DFL was obtained for the triaxial compression tests conducted under a confining pressure $\sigma_{c}=200\;\mathrm{kPa}$ for sand−cement mixtures with different amounts of cement and different cement−void ratios (Figure 8).

This means that for these sand−cement mixtures, the DFL does not depend on the amount of cement or the cement−void ratio.

### 4.2. Residual Granitic Soil with Different Cement

**Figure 9:**
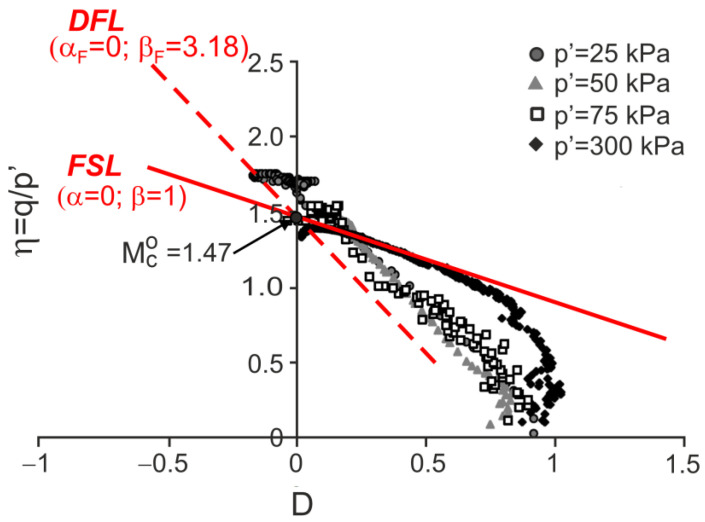
Stress ratio–dilatancy relationships for the drained triaxial compression of decomposed granitic residual soil (adapted from [7]). Reprinted from Publication Proceedings of the 15th European Conference on Soil Mechanics and Geotechnical Engineering, Part 2, Cruz, N.; Viana da Fonseca, A.; Rodrigues, C., The influence of microfabrics in bonded soils behaviour, based in laboratorial comparison of artificially and naturally cemented specimens, 173–178, Copyright (2022), with permission from IOS Press. The publication is available at IOS Press through http://doi.org/10.3233/978-1-60750-801-4-173.

**Figure 10:**
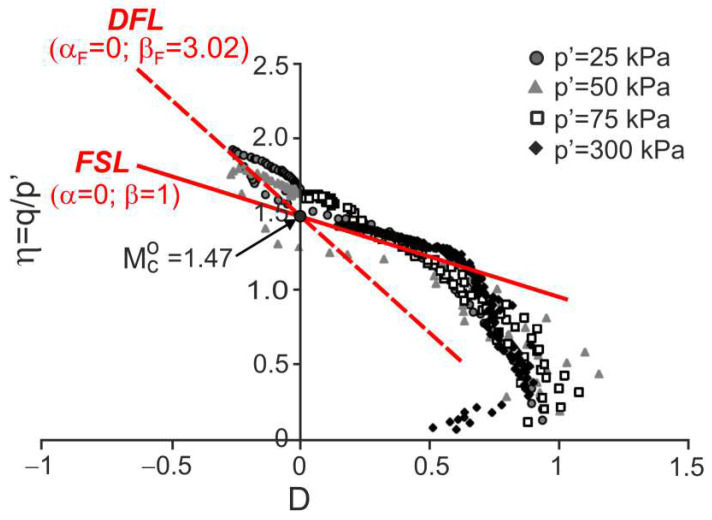
Stress ratio–dilatancy relationships for the drained triaxial compression of granitic residual soil with 1% of cement 52.5R (adapted from [7]). Reprinted from Publication Proceedings of the 15th European Conference on Soil Mechanics and Geotechnical Engineering, Part 2, Cruz, N.; Viana da Fonseca, A.; Rodrigues, C., The influence of microfabrics in bonded soils behaviour, based in laboratorial comparison of artificially and naturally cemented specimens, 173-178, Copyright (2022), with permission from IOS Press. The publication is available at IOS Press through http://doi.org/10.3233/978-1-60750-801-4-173.

**Figure 11:**
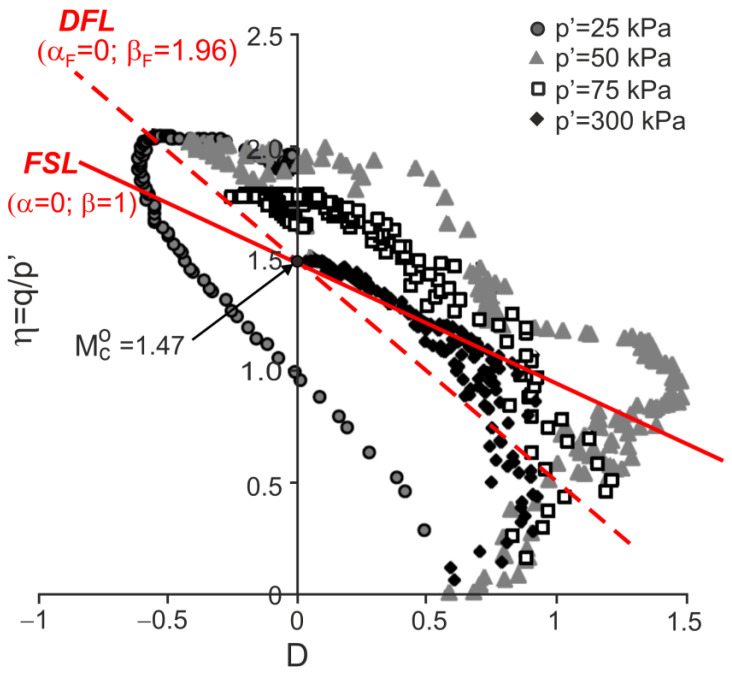
Stress ratio–dilatancy relationships for the drained triaxial compression of granitic residual soil with 2% of cement 32.5N (adapted from [7]). Reprinted from Publication Proceedings of the 15th European Conference on Soil Mechanics and Geotechnical Engineering, Part 2, Cruz, N.; Viana da Fonseca, A.; Rodrigues, C., The influence of microfabrics in bonded soils behaviour, based in laboratorial comparison of artificially and naturally cemented specimens, 173-178, Copyright (2022), with permission from IOS Press. The publication is available at IOS Press through http://doi.org/10.3233/978-1-60750-801-4-173.

**Figure 12:**
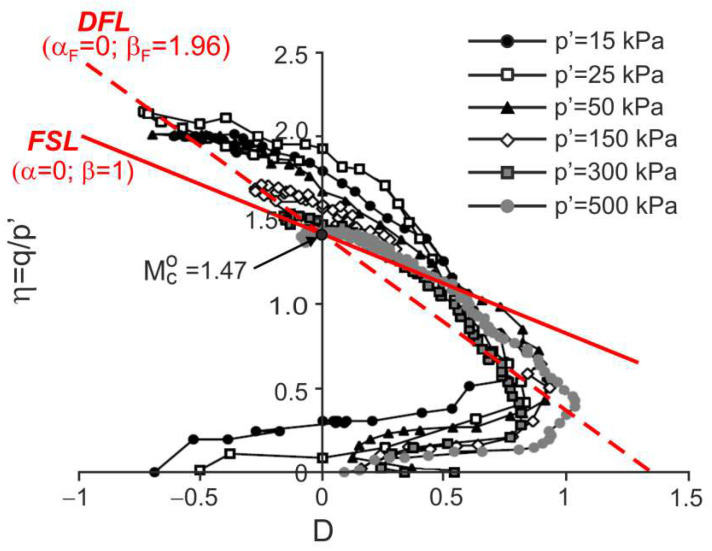
Stress ratio–dilatancy relationships for the drained triaxial compression of natural granitic residual soil (adapted from [7]). Reprinted from Publication Proceedings of the 15th European Conference on Soil Mechanics and Geotechnical Engineering, Part 2, Cruz, N.; Viana da Fonseca, A.; Rodrigues, C., The influence of microfabrics in bonded soils behaviour, based in laboratorial comparison of artificially and naturally cemented specimens, 173-178, Copyright (2022), with permission from IOS Press. The publication is available at IOS Press through http://doi.org/10.3233/978-1-60750-801-4-173.

Drained triaxial compression tests on natural and remoulded residual granitic soil and mixtures of this remoulded soil with admixtures of Portland cement 52.5R and 32.5N were carried out by Cruz [28] and Cruz et al. [7]. The stress ratio versus dilatancy obtained in the tests is shown in Figure 9, Figure 10, Figure 11 and Figure 12.

For all the tests, the characteristic points of shearing can be easily identified (they are not shown in the figures for clarity). Therefore, the DFLs can be drawn in the η−D planes. The DFLs intersect the vertical axes at $\eta =M_{c}^{o}=1.47$, which means that for all the tested materials, the critical state friction angle $\phi^{o}=36.2{}^{\circ}$. The DFL slopes, determined by $\beta_{F}$, are $\beta_{F}=3.18$ for destructured soil, $\beta_{F}=3.02$ for destructured soil with a 1% admixture of 52.5R cement, and $\beta_{F}=1.96$ for destructured soil with a 2% admixture of 32.5N cement and natural residual granitic soil. The frictional state lines (FSL) with α=0 and β=1 representing the failure states of unbonded quartz sands [18] are also shown in Figure 9. It is unclear why the DFL slope for the destructured granitic residual soil without cement, a less structured soil, is higher than those for artificially bonded and structured natural soils.

## 5. Conclusions

(1) For bonded soils, geomaterials with high stiffness, the elastic parts of the strains in global strains are small, and it can be assumed that the dilatancy and plastic dilatancy are equal.
(2) The stress−dilatancy behaviour of naturally and artificially bonded soils in all stages of plastic flow can be approximated by the linear stress−dilatancy equation of the Frictional State Concept.
(3) The failure and dilatant failure states are not equivalent for many triaxial compression tests of bonded soils. Dilatant failure states are more characteristic than failure states for these geomaterials.
(4) The dilatant failure state line in the η−D plane is defined by $\alpha_{F}=0$ and $\beta_{F}$, and intersects the vertical axis at the point $\eta =M_{c}^{o}$, defining the critical frictional state angle ($\phi^{o})$. The value of parameter $\beta_{F}>1$ for artificially bonded soils.
(5) The parameters α and β for different stages of shear are functions of the quantity of the cement admixture and soil type. The frictional critical state angle ($\phi^{o})$ does not depend on the amount of cement for the analysed artificially bonded soils.
(6) The variety of the stress−strain behaviours of bonded soils can be described by the stress−dilatancy relationship, but the mechanics of this behaviour cannot be explained by the frictional state concept. The complex stress−strain behaviour of bonded soils should be further investigated experimentally and theoretically.

## Data Availability

Not applicable.
